# Overall intact cognitive function in male X-linked adrenoleukodystrophy adults with normal MRI

**DOI:** 10.1186/s13023-019-1184-4

**Published:** 2019-09-14

**Authors:** Noortje J. M. L. Buermans, Sharon J. G. van den Bosch, Irene C. Huffnagel, Marjan E. Steenweg, Marc Engelen, Kim J. Oostrom, Gert J. Geurtsen

**Affiliations:** 1 Department of neuropsychology, Emma Children’s Hospital, Amsterdam UMC, University of Amsterdam, Amsterdam, The Netherlands; 2 Department of Medical Psychology, Amsterdam Neuroscience, Amsterdam UMC, University of Amsterdam, PO Box 22660, 1105 AZ Amsterdam, The Netherlands; 3 Department of Pediatric Neurology, Emma Children’s Hospital, Amsterdam UMC, University of Amsterdam, Amsterdam, The Netherlands

**Keywords:** X –linked adrenoleukodystrophy, Natural history studies, MRI, Leukodystrophies, Peroxisomes, Neuropsychological assessment

## Abstract

**Background:**

Men with the hereditary peroxisomal disorder X-linked adrenoleukodystrophy (ALD) are at risk of developing inflammatory demyelinating lesions in the brain. In the absence of inflammatory (post-contrast enhancing) lesions on MRI cognitive function is considered spared, but some form of cognitive dysfunction may nevertheless be present. The aim of this cross-sectional study was to characterize cognitive functioning of ALD men with no or minimal MRI abnormalities, which will define cognitive functioning in this category of patients.

**Methods:**

A neuropsychological battery covering a broad range of cognitive domains, including language, verbal and non-verbal memory, visuoconstruction, executive functioning, and psychomotor speed, was used. Means and proportions of borderline and impaired T scores ≤36 were compared to the standardized norm group and a qualitative case-by-case analysis was performed for participants with T scores ≤36 within ≥2 domains. Patients with MRI abnormalities that were extensive (Loes score > 3) or showed enhancement post-contrast were excluded.

**Results:**

Thirty-three men participated (median age 44 years, range 19–71). Mean performance on verbal fluency was poorer in patients (45.70 ± 8.85 patients vs. 50 ± 10 standardized norm group, *p* = 0.009), as was the percentage of borderline and impaired scores on visuoconstruction (Beery VMI: 19% patients vs. 8% standardized norm group, *p* = 0.02; RCFT copy: 81% patients vs. 2% standardized norm group, *p* < 0.0005) and mental reaction time during a complex decision task (18% patients vs. 8% standardized norm group, *p* = 0.055). Moreover, 9/33 (27.3%) patients had T scores ≤36 within ≥2 domains.

**Conclusions:**

Given the heterogeneous pattern of mostly borderline scores cognitive functioning seems not impaired in the vast majority of adult ALD males with no or minimal MRI abnormalities. However, borderline to impaired cognitive dysfunction was present in 27.3%, with the majority being borderline scores. Longitudinal studies will have to determine if this reflects early cerebral disease under the detection limit of MRI.

## Introduction

Boys and men with the hereditary peroxisomal disorder X-linked adrenoleukodystrophy (ALD) are at risk of developing inflammatory demyelinating lesions in the brain (‘cerebral ALD’) [1]. Although all patients have an *ABCD1* mutation, only some develop inflammatory brain lesions and predicting who is not possible. Untreated the brain lesions are usually rapidly progressive and cause severe disability and death. Haematopoietic stem cell transplantation stabilizes lesions if performed in an early stage of the disease [2–5]. Although overall cognitive functioning is considered spared as long as there are no inflammatory lesions on MRI [6], some form of cognitive dysfunction may be present in patients without lesions [7]. Indeed, in ALD boys with no or minimal MRI abnormalities overall cognitive functioning was intact, but some dysfunction in visual perceptual, visuomotor or visual reasoning skills and verbal skills was present [8–10]. Similarly, in 52 adult ALD men with no or minimal MRI abnormalities verbal and visual memory, psychomotor speed, and visuoconstruction were impaired in some of them, however these findings were based on a cognitive test battery that didn’t fully cover all cognitive functions and have not been confirmed in later studies [7]. The detected cognitive dysfunction could reflect functional abnormalities of the white matter caused by the underlying genetic defect or perhaps even very early signs of inflammatory demyelinating lesions under the detection limit of structural MRI.

The purpose of this cross-sectional study was to characterize cognitive functioning of male ALD adults with no or minimal MRI abnormalities. This will define cognitive functioning in this category of ALD patients and provide directives on the neuropsychological requirements of ALD patients during the course of the disease.

## Methods

### Participants

In this cross-sectional study Dutch ALD patients from the ongoing prospective natural history study (‘The Dutch ALD cohort’) [11] were approached to participate between June 2016 and February 2017. Men aged 18 years or older with available (3.0 Tesla) MRI results were eligible for inclusion. Men with co-morbidity that would interfere with the interpretation of neuropsychological testing results or with MRI abnormalities that were extensive or showed enhancement post-contrast were excluded from participation. MRI abnormalities were considered extensive if the Loes score was over three. The Loes score is an ALD MRI score, which rates the severity of white matter lesions and ranges from 0 (normal) – 34 (abnormal) [12]. MRI’s were scored by two independent physicians (IH and MS). The physicians were blinded to the neuropsychological test results. If the MRI scores varied, they were debated until a consensus was reached. All white matter abnormalities were scored, unless they were small, round and highly aspecific. Atrophy was solely scored in the presence of white matter abnormalities. White matter abnormalities were further categorized into three categories based on their distribution and shape: ALD lesions, vascular lesions and other lesions. Confluent white matter lesions with increased signal intensity on T2-weighted and FLAIR images were considered ALD lesions, whereas diffuse irregular white matter lesions with punctate foci were considered vascular lesions. Lesions that did not appear ALD like or vascular were labelled as other lesions.

### Standard protocol approvals, registrations, and patient consents

The study protocol was approved by the local Institutional Review Board (METC 2016_012). Written informed consent was obtained from all participants.

### Procedure

Participation included one comprehensive neuropsychological assessment and took place at the Amsterdam UMC in Amsterdam, The Netherlands. A standardized neuropsychological battery was composed to examine cognitive (dys)function across different cognitive domains. Test results were compared to Dutch standardized norm groups (*N* = 276–1600), that corrects for age, education level and/or gender. The neuropsychological tests as well as the Dutch standardized norm groups are frequently used in neuropsychological practice and research (Table 1). The duration of the neuropsychological assessment was approximately two hours and was administered by a well-trained neuropsychologist (in training) in a single session.

**Table 1:** Neuropsychological assessment battery

Cognitive domain	Test	Corrected for	N
Language	COWAT letter fluency test, Dutch version (DAT) [13] Vocabulary and Similarities subtests of the WAIS-IV [14]	Education level Age	570 1009
Verbal memory	Rey AVLT, Dutch version [15, 16]	Age, education level and gender	847
Non-verbal memory	Visual Reproduction subtest of the WMS-IV [17]	Age	1188
Visuoconstruction	RCFT-copy subtest [18] Beery VMI-VI [19]	Age Age	601 1021
Executive functioning	TMT of the Halstead–Reitan Battery [20] Stroop Color and Word Test [21]	Age, education level Age, education level and gender	478 803
Psychomotor speed	Subtests S1 and S3 of the VTS (Version 8.2) [22]	Age^a^	276/981
Subjective assessment	BRIEF-A self-report [23]	No corrections	1600

Abbreviations: *AVLT* Auditory Verbal Learning Test, *BRIEF-A* Behavior Rating Inventory of Executive Function - Adult Version, *COWAT* Controlled Oral Word Association Test, *RCFT* Rey Complex Figure Test, *TMT* Trail Making Test, *VMI* Visual-Motor Integration, *VTS* Vienna Test System, *WAIS* Wechsler Adult Intelligence Scale, *WMS* Wechsler Memory Scale. ^a^S1 only above or below 51 years

N number of persons in standardised norm group

### Statistical analyses

The data was analysed with IBM SPSS statistics version 24 (IBM Inc.) and MedCalc Statistical Software version 17.9 [24]. Raw scores were converted to standardized T scores. T scores are commonly used for neuropsychological normative data and are comparable with *z* scores. Like *z* scores, T scores are a standard score, which are calculated with standardized norm group corrected for age, gender and/or educational level. T scores have a mean of 50 and a standard deviation (SD) of 10, whereas *z* scores have a mean of 0 and a SD of 1 (e.g., *z* = − 1 equals T = 40; *z* = 1 equals, T = 60). Higher T scores represent better cognitive function. For this exploratory study, we considered T scores between 30 and 36 (− 2 SD to − 1.5 SD below standardized norm group mean; ‘borderline’) to indicate borderline scores and T scores of 29 and lower (> − 2 SD below standardized norm group mean; ‘extremely low scores’) as impairment [25, 26]. An exception was the RCFT-copy subtest. On this test the maximum T score is 40. Therefore, RCFT-copy T scores were categorized into ‘Normal’ (T scores ranging from 38 to 40), ‘borderline’ (T scores ranging from 30 to 37) and ‘impaired’ (T scores ≤29) [18, 27].

The Shapiro-Wilk test was used to test the assumption of normality. The distribution of the educational level of the participants, based on the Dutch educational classification scale of Verhage (1983) [28], was compared to the male Dutch population with a chi-square goodness-of-fit test [29]. For comparisons of the neuropsychological test scores between groups first one-sample *t* tests (normally distributed continuous data) or one-sample Wilcoxon signed rank tests (non-normally distributed continuous data) were used to compare the average T scores with the average T score of the standardized norm group (50 ± 10). Then, one-proportion z-tests were used to compare the proportion of borderline (T scores between 30 and 36) to impaired scores (T scores ≤29) on neuropsychological tests within our cohort with the proportion borderline and impaired scores that occur in the standardized norm group (8%) [25]. Lastly, the distribution of the categorical RCFT copy subtest scores was analysed with chi-square goodness-of-fit tests. The expected frequency was set to a normal distribution in the population. *P* values < 0.05 (two-tailed) were defined as statistically significant.

Borderline scores in itself are no indication of impairment unless there is a clear decrease over time and the pattern of borderline scores is consistent. Impaired scores reflect impairment [26].

To evaluate the possible effect of minor MRI abnormalities the comparisons of neuropsychological test scores with the standardized norm group were performed four times. First, including all patients (primary analysis). Second, including only patients with a completely normal MRI (subgroup analysis 1). Third, including patients with a completely normal MRI and patients with ALD lesions (Loes score ≤ 3) (subgroup analysis 2) and fourth, including patients with a completely normal MRI and patients with vascular lesions (subgroup analysis 3).

Last, a qualitative case-by-case analysis was performed to see which patients had borderline or impaired scores (T scores ≤36) within 2 or more cognitive domains. Univariate logistic regression analyses were used to evaluate the effect of age or the presence of MRI abnormalities on the qualitative case-by-case analysis outcome (T scores ≤36 within 2 or more cognitive domains yes/no).

## Results

### Demographics

Of the 39 adult men with ALD participating in the natural history study, 4 patients had a Loes score > 3 and one patient had non-ALD related intellectual disability. The remaining 34 eligible patients were approached for participation, of whom 33 agreed. Certain test battery elements were excluded per participant due to poor eyesight (< 20%) and colour-blindness (TMT, Stroop, Beery, RCFT-copy) in one case; solely colour-blindness (Stroop II, III, III/II) in one case; an essential tremor (TMT A, TMT B, Beery VMI, RCFT-copy, VTS) and daily benzodiazepine use (VTS) in 2 cases; and, inconsistent, extreme negative or unreliable self-report scores on the BRIEF-A in 3 cases. Median age was 44 years (range 19–71). The most frequent education levels were secondary vocational education (14/33) and (higher) secondary education or university of applied sciences (14/33). The distribution of educational levels was significantly different in comparison to the male Dutch population (x^2^ (4) = 11.806, *p* = 0.019). The proportion of patients with secondary vocational education and higher secondary education was higher than in the Dutch population, and the proportion of primary or lower vocational education and university bachelor or masters degree was lower (Table 2). White matter lesions on MRI were present in 18/33 (54.5%) patients, including ALD lesions (*n* = 4), vascular lesions (*n* = 12) and other types of lesions (*n* = 2). The other lesions included a lesion suggestive of an old cerebral contusion in one patient and aspecific white matter lesions in another (Table 2). In the patients with vascular lesions the maximum Fazekas grade was one [30].

**Table 2:** Patient characteristics

	Number of patients (%) or median (range)	Number of Dutch male population (%) or median (range)^a^
N (Total)	33	5,446,000
Age in years	44 (19–71)	(15–100)
Education		
1: < Primary education	0 (0)	0 (0)
2: Primary education	0 (0)	464,000 (8.5)
3: < Lower vocational education	1 (3)	302,000 (5.5)
4. Lower vocational education	2 (6.1)	1,290,500 (23.7)
5. Secondary vocational education	14 (42.4)	1,272,500 (23.4)
6. (higher) Secondary education or university of applied sciences	14 (42.4)	1,507,000 (27.7)
7. University bachelor or masters degree.	2 (6.1)	610,000 (11.2)
MRI		
Normal MRI (no lesions)	15 (45.5)	
MRI with ALD lesions	4 (12.1)	
MRI with vascular lesions	12 (36.4)	
MRI with other lesions	2 (6.1)	

Education scores are measured according to Verhage’s (1983) educational classification system [28]

^a^ Data from the Dutch male population (2016) derived from CBS (Central Statistical Office Netherlands) and is available online [28]

### Cognitive functioning - primary analyses (including all patients)

First average T scores were compared to standardized norm group values. The mean T score for the letter fluency test (45.70 ± 8.85) was statistically significant lower in patients with a difference of 4.30 (95% confidence interval (CI), − 7.44 to – 1.16), *t* (32)*=* − 2.793, *p* = 0.009). The group means and medians of all other tests with continuous measures were not significantly lower from the mean of the standardized norm group (Table 3). Second, percentages of borderline and impaired T scores (≤36) were compared with the percentage in the standardized norm group (8%) (Table 4). The percentage borderline and impaired T scores on the Beery VMI in the patients (19%) was significantly higher than in the standardized norm group (*z* = 2.33, *p* = 0.02). The percentage borderline and impaired T scores on the VTS S3 RT (18%) tended to be higher than in standardized norm group (*z* = 1.92, *p* = 0.055). Last, for the RCFT copy subtest results were normal in 2/31, suboptimal in 4/31 and impaired in 25/31. Scores were not distributed as expected (x^2^ (1)=803.107, *p* < 0.0005).

**Table 3:** T scores of adult male ALD patients compared to the standardized norm group (mean = 50)

Neuropsychological test per cognitive domain	N	Mean ± SD or median (range)	*p* value
Language			
Letter fluency	33	45.70 ± 8.85	0.009*
Similarities**	33	50 (30–58)	0.050
Vocabulary	33	47.18 ± 8.00	0.051
Verbal memory			
REY AVLT IR	33	46.97 ± 10.41	0.104
Rey AVLT DR	33	49.24 ± 10.05	0.668
Rey AVLT DR/IR	33	51.52 ± 7.72	0.268
Non-verbal memory			
WMS VR IR**	32	50 (33–72)	0.421
WMS VR DR	32	55.44 ± 9.00	0.002*
WMS VR recognition**	32	58 (35–58)	0.001*
Visuoconstruction			
Beery VMI**	31	49 (19–64)	0.193
Executive functioning			
TMT A	31	57.29 ± 12.95	0.004*
TMT B	31	53.55 ± 11.18	0.087
TMT B/A	31	49.81 ± 9.32	0.909
Stroop I**	32	48 (28–85)	0.172
Stroop II	31	49.94 ± 10.34	0.973
Stroop III	31	53.23 ± 11.14	0.117
Stroop III/II	31	55.42 ± 10.59	0.008*
BRIEF A**	30	51 (36–83)	0.186
Psychomotor Speed			
VTS-S1-RT	29	57.66 ± 12.04	0.002*
VTS-S1-MT	29	58.10 ± 11.95	0.001*
VTS-S3-RT	28	46.89 ± 10.72	0.137
VTS-S3-MT	28	51.71 ± 10.92	0.414

T scores of patients were compared to the standardized norm group. The distribution of T scores of the standardized norm group has a mean of 50, with a standard deviation of 10

Abbreviations: * = *p* < 0.05 (two-tailed); ** = Non-normally distributed data; *BRIEF-A* Behavior Rating Inventory of Executive Function - Adult Version, *DR* delayed recall, *IR* immediate recall, *M* mean, *N* number of patients, *p p* value, *RCFT* Rey Complex Figure Test, *SD* standard deviation, *TMT* Trail Making Test, *VMI* Visual-Motor Integration, *VTS-S1-MT* Vienna Test System Subtest 1 Motor Reaction Time, *VTS-S1-RT* Vienna Test System Subtest 1 Mental Reaction Time, *VTS-S3-MT* Vienna Test System Subtest 3 Motor Reaction Time, *VTS-S3-RT* Vienna Test System Subtest 3 Mental Reaction Time, *WMS VR* Wechsler Memory Scale Visual Reproduction

Normally distributed data is presented as mean ± standard deviation (range). Data that was not normally distributed is presented as median (range)

**Table 4:** Frequencies of T scores and borderline and impaired T scores (≤ 36) from adult male ALD patients compared to the percentage in the standardized norm group (8%)

		N	Test T score							Z	*p*	95% CI
			≥ 70	64–69	57–63	44–56	37–43	30–36 (%)	≤ 29 (%)			
Language	Letter fluency	33	0	0	4	16	8	4 (12.1	1 (3)	1.514	0.130	5.11–31.90
	Similarities	33	0	0	6	13	11	3 (9)	0	0.231	0.818	1.92–24.33
	Vocabulary	33	0	0	5	16	10	2 (6)	0	0.411	0.681	0.74–20.23
Verbal memory	REY AVLT IR	33	0	0	6	16	6	2 (6)	3 (9)	1.514	0.130	5.11–31.90
	Rey AVLT DR	33	0	2	7	11	11	2 (6)	0	0.411	0.681	0.74–20.23
	Rey AVLT DR/IR	33	0	2	8	19	4	0	0	1.694	0.090	0.00–10.58
Non-verbal memory	WMS VR IR	32	2	2	4	18	5	1 (3)	0	1.015	0.310	0.08–16.22
	WMS VR DR	32	4	1	10	14	3	0	0	1.668	0.095	0.00–10.89
	WMS VR recognition	32	0	0	18	11	2	1 (3)	0	1.015	0.310	0.08–16.22
Visuoconstruction	Beery VMI	31	0	1	2	19	3	3 (9.7)	3 (9.7)	2.329	0.020*	7.45–37.47
Executive functioning	TMT A	31	5	6	3	13	3	1 (3.2)	0	0.979	0.328	0.08–16.71
	TMT B	31	4	2	6	14	3	2 (6.5)	0	0.318	0.750	0.79–21.42
	TMT B/A	31	1	1	6	16	5	2 (6.5)	0	0.318	0.750	0.79–21.42
	Stroop I	32	2	1	1	19	6	2 (6.3)	1 (3,1)	0.288	0.774	1.98–25.03
	Stroop II	31	2	2	2	16	7	1 (3.2)	1 (3.2)	0.318	0.750	0.79–21.42
	Stroop III	31	3	2	4	15	5	2 (6.5)	0	0.318	0.750	0.79–21.42
	Stroop III/II	31	3	3	9	12	3	1 (3.2)	0	0.979	0.328	0.08–16.71
	BRIEF A	30	3	3	3	18	2	1 (3.3)	0	0.943	0.346	0.08–17.21
Psychomotor speed	VTS-S1-RT	29	4	3	9	9	3	0	1 (3.4	0.903	0.366	0.09–17.77
	VTS-S1-MT	29	4	5	6	12	2	0	0	1.588	0.112	0.00–11.94
	VTS-S3-RT	28	1	2	3	9	8	5 (17.8)	0	1.923	0.055	6.07–36.90
	VTS-S3-MT	28	0	4	6	10	6	2 (7.1)	0	0.168	0.8668	0.88–23.50

The frequency of borderline and impaired T scores from patients, defined as the percentage of T scores ≤36, were compared to the percentage of borderline and impaired T scores of the standardized norm group. As T scores of the standardized norm group follow a normal distribution, the percentage of borderline and impaired T scores in the standardized norm group equals 8%. Abbreviations: * *p* < 0.05 (two-tailed); *CI* confidence interval from the percentage borderline and impaired T scores, *DR* delayed recall, *IR* immediate recall, *N* number of patients, *p p* value, *Rey VLT* Rey Verbal Learning Test, *TMT* Trail Making Test, *VMI* Visual-Motor Integration, *VTS-S1-MT* Vienna Test System Subtest 1 Motor Time, *VTS-S1-RT* Vienna Test System Subtest 1 Mental Reaction Time, *VTS-S3-MT* Vienna Test System Subtest 3 Motor Time, *VTS-S3-RT* Vienna Test System Subtest 3 Mental Reaction Time, *WMS VR* Wechsler Memory Scale Visual Reproduction, *Z* z-statistic

### Cognitive functioning - subgroup analyses

Besides comparing test scores of all patients with the standardized norm group, three subgroup analyses were performed to evaluate the possible effect of minor MRI abnormalities. Again, average T scores were compared to standardized norm group values. For all subgroup analyses no additional significantly lower mean T scores were detected.

In addition, percentages of borderline and impaired T scores (≤36) were compared with the percentage in the standardized norm group (8%). When solely including subgroup 1 (patients with a completely normal MRI) the percentage borderline and impaired T scores on the VTS-S3-RT became significantly higher than in the standardized norm group (*p* = 0.045). When including subgroup 2 (patients with a completely normal MRI and patients with ALD lesions with a Loes score ≤ 3) the percentage borderline and impaired T scores on the letter fluency test became significantly higher in comparison to the standardized norm group (*p* = 0.0032). When including subgroup 3 (patients with a completely normal MRI and patients with minor vascular lesions) the percentage borderline and impaired T scores on the VTS-S3-RT became significantly higher than the standardized norm group (*p* = 0.021).

For the RCFT copy test subgroup analyses were not possible due to insufficient numbers per category.

### Case-by-case analysis

Case-by-case analyses revealed that 6/33 (18.2%) patients had borderline to impaired T scores (T scores ≤36) across 2 cognitive domains and 3/33 (9%) patients had borderline to impaired scores across 3 cognitive domains. Of these 9 patients showing deficits in 2 or more cognitive domains, 5 had a completely normal MRI, 2 had ALD lesions, and 2 had vascular lesions. Of the 6 patients with 2 affected cognitive domains, psychomotor speed was most prevalent (4/6), followed by executive functioning and visuoconstruction (3/6) and language and non-verbal memory (2/6). In all patients with 3 affected cognitive domains language was present, and verbal memory and executive functioning in most (2/3). None of the patients had borderline to impaired scores on all three tests on which performance of our group was reduced, i.e. letter fluency test, VTS-S3-RT and Beery VMI, nor was another neuropsychological test profile detected consistent among all 9 patients. In the logistic regression neither age (coefficient = − 0.021, standard error 0.025, *p* = 0.397) nor the presence of MRI abnormalities (coefficient = − 0.56, standard error = 0.788; *p* = 0.478) were statistically significant predictors for the presence of borderline T scores across 2 or more cognitive domains. Only one patient (3%) scored in the impaired range (T scores ≤29 across 2 cognitive domains).

## Discussion

This study confirms that overall cognitive functioning of adult male ALD patients with a normal MRI or minimal MRI abnormalities seems intact, but that significant individual variability exists in 27.3%. The majority (24.2%) show borderline scores (T-score > 29 ≤ 36; see Table 4) and only 3% show an impairment.

Although overall cognitive functioning was intact, subtle cognitive deficits were detected when comparing the average and the distribution of test scores of our patient group to standardized norm group on visuoconstructive tasks (Beery VMI and RCFT copy subtest; 6/31), mental reaction time measured during a complex decision task (VTS-S3-RT; 5/28) and on a verbal fluency task (letter fluency test; 5/33). Moreover, qualitative case-by-case analyses revealed that 9/33 (27.3%) patients had borderline or impaired performances across 2 or more cognitive domains. However, the distribution of these lower scores were heterogeneous over the cognitive domains and contradictive. For instance a borderline score on a decision psychomotor speed test while another speed and executive tests were normal. Additional follow-up studies, however, are necessary to confirm if this borderline to impaired performance reflects an impaired neuropsychological profile and may represent a risk profile for cerebral X-ALD.

As previous findings in the study of Edwin et al. (1996) were limited [7], this study measured cognitive functions more broadly and used two or more (sub)tests for each cognitive domain (visuoconstruction, executive functioning, psychomotor speed, memory and language). Furthermore, this study used a 3 T MRI that has a higher resolution and can detect smaller lesions than the 1.5 T MRI that was used in the study of Edwin et al. [7]. Our findings support the findings of Edwin et al. (1996) as patients showed subtle cognitive deficits on visuoconstructive functioning [7]. Besides, our study showed a weaker verbal fluency, that was also seen in a previous study on asymptomatic ALD boys [7, 10]. Moreover, Edwin et al. (1996) reported impaired verbal fluency relatively early in the cerebral manifestation of the disease [7]. Likewise, we replicated the deficits within psychomotor speed reported by Edwin et al. (1996) [7], although in our study this deficit was only present on a task measuring mental reaction during a more complex decision. This difference could be caused by the task used, as Edwin et al. (1996) assessed psychomotor speed with the Grooved Pegboard task, which relies highly on fine fingertip dexterity and measures motor speed and we administered the Vienna Test System [7, 31], which makes a distinction in motor and mental reaction time [22]. Perhaps ALD patients have difficulties in decision-making in a more complex situation (e.g. when more stimuli need to be interpreted instead of a single stimulus), but gross motor function of the arm is still intact. Furthermore, as reported by others [7, 8, 10], executive functioning seems intact, although verbal fluency and mental reaction time during a complex decision task were slightly impaired in our cohort, which also highly rely on executive abilities [13, 22].

In some patients borderline to impaired scores are present even in the absence of a significant white matter lesion load on MRI. Hypothetically, functional abnormalities of the white matter caused by mutations in the *ABCD1* gene – the underlying genetic defect in ALD - or very early signs of inflammatory demyelinating lesions under the detection limit of MRI might already be present in these patients [32]. Quantitative neuroimaging studies using magnetic resonance spectroscopy (MRS) have shown alterations in metabolite levels in normal appearing white matter of ALD patients [33–35]. In addition, as the inflammatory cerebral manifestation of ALD manifests itself mostly in the splenium of the corpus callosum extending into the parieto-occipital white matter, this could reflect the cognitive deficits we found in visuoconstruction and mental reaction time [36, 37]. Less often white matter lesions are observed in the genu of the corpus callosum and progress to frontal white matter [6, 12, 34, 38], which could represent the somewhat affected verbal fluency. Moreover, like the splenium and the parieto-occipital white matter, the frontal brain regions are also involved in mental reaction time [36]. However, due to the small number of patients in this study these speculations need to be confirmed in future studies.

Although this study reports valuable data on the cognitive functioning of adult male ALD patients with no or minor MRI abnormalities, various uncertainties in the interpretations of our results remain. While this study is one of the larger ALD cohort studies, the size of the sample was still small and we had to exclude some test battery elements in some patients. This reduces statistical power, makes proper adjustment for confounders impossible and caution in the interpretation of our results is warranted as this might have caused selection bias and type II errors (not finding impairment when they are actually there) cannot be ruled out. Despite a relatively small sample size, the subgroup analyses do suggest that the sample was representative for other ALD patients. The degree of cognitive dysfunction in ALD patients has been correlated to lesion load on MRI [6, 7, 10, 39], and in our cohort 4 patients had ALD lesions on MRI and 12 minor vascular lesions (maximum Fazekas grade 1). Indeed, vascular lesions are associated with cognitive dysfunction [40]. But, vascular lesions are frequently present in the general population and therefore probably also in participants included in the standardized norm group. Results remained the same when excluding the subgroups with MRI abnormalities. Furthermore, 5/9 patients with borderline to impaired performances across 2 or more cognitive domains had a completely normal MRI. In addition, regression analyses confirmed that the presence of MRI abnormalities was not a significant predictor of the presence of T scores ≤36 within 2 or more cognitive domains. Moreover, although the distribution of educational levels differed from the general Dutch population, test scores were adjusted for education level reducing possible selection bias. Meanwhile, it remains unclear if the diminished RCFT-copy test results reflects clinically relevant information on visuoconstruction, as criterion validity (how well test results are related to a clinical outcome) of this test is marginal [41]. This study used Dutch standardized norm groups (*N* = 276–1600). The advantage of such large reference groups is the possibility to correct for the influence of age, education level and/or gender. This is not possible in often used smaller case control or control groups.

A major shortcoming of this study is that only cross-sectional data from the cohort is available at this time with individual data at one time point for patients across a wide range of ages. This neglects the temporal nature of X-ALD and the possibility of within individual age-related changes over the life time. In addition, multivariable analyses were not possible using the current methods. Follow-up is needed and is planned in order to monitor cognitive functioning within this cohort and to evaluate if alterations across these cognitive domains precede the onset of the cerebral manifestation of the disease. If the detected abnormalities persist and progress, cognitive functioning can have predictive value superior to currently used structural MRI. Identification of patients with the cerebral manifestation remains important as illustrated by recent work of Pierpont et al. [42]. Even in boys with a relatively low lesion load on MRI (Loes score ≤ 4.5) severe cognitive impairments were detected 4 years after haematopoietic stem cell transplantation [42].

In conclusion, this study shows that cognitive functioning seems intact in adult male ALD patients with no or minimal MRI abnormalities. However, there are indications of borderline scores and cognitive impairments in a subgroup of patients affecting the domains of visuoconstruction, verbal fluency, mental reaction time and possibly executive functioning. The necessity for prospective studies remains to assess the relevance of this deviant scores and if neuropsychological assessment – perhaps in combination with advanced MRI techniques - can detect the onset of cerebral inflammatory demyelination before structural MRI.

## Data Availability

The datasets used and/or analysed during the current study are available from the corresponding author on reasonable request.
